# Machine learning-based prediction model for hypoalbuminemia in ICU patients with venous thromboembolism and acute kidney injury

**DOI:** 10.3389/fmed.2026.1922098

**Published:** 2026-10-13

**Authors:** Zelong Qian, Guoqing Li, Junjian Tang

**Affiliations:** Affiliated Hospital of Jiangnan University, Jiangnan University, Wuxi, Jiangsu, China

**Keywords:** acute kidney injury, hypoalbuminemia, intensive care unit, machine learning, prediction model, venous thromboembolism, XGBoost

## Abstract

**Background:**

Hypoalbuminemia is a prevalent and critical complication among intensive care unit (ICU) patients with venous thromboembolism (VTE) and acute kidney injury (AKI), significantly associated with increased mortality and prolonged hospitalization. However, reliable tools for early prediction of hypoalbuminemia in this high-risk population remain limited. This study aimed to develop and validate a machine learning-based prediction model for hypoalbuminemia in ICU patients with coexisting VTE and AKI.

**Methods:**

In this retrospective study, data from 585 ICU patients with VTE and AKI were analyzed. Patients were divided into a training cohort (*n* = 409) and a validation cohort (*n* = 176). Least absolute shrinkage and selection operator (LASSO) regression with standardization was applied for feature selection. Five machine learning models-logistic regression (LR), random forest (RF), extreme gradient boosting (XGBoost), light gradient boosting machine (LightGBM), and support vector machine (SVM)-were developed and evaluated using 5-fold cross-validation. Model performance was assessed by the area under the receiver operating characteristic curve (AUC), accuracy, precision, recall, and F1-score. Decision curve analysis (DCA), SHapley Additive exPlanations (SHAP), and feature importance were performed for the optimal model. The performance of the best machine learning model was compared with conventional ICU severity scores (APSIII, LODs, and OASIS).

**Results:**

The standardized LASSO regression identified ten optimal predictors: creatinine (cr), age, tumor/cancer, body mass index (BMI), low-density lipoprotein (LDL), urine output, D-dimer (D_D), Charlson comorbidity index, anticoagulant use, and alanine aminotransferase (ALT). Among the five models, XGBoost achieved the best predictive performance with a validation AUC of 0.882 [95% confidence interval (CI): 0.832–0.924], followed by RF (AUC = 0.878), LightGBM (AUC = 0.870), LR (AUC = 0.838), and SVM (AUC = 0.662). The 5-fold cross-validation AUC for XGBoost was 0.901 ± 0.042, with minimal overfitting (CV-test difference = −0.019). Decision curve analysis demonstrated favorable clinical net benefit of the XGBoost model across a wide range of threshold probabilities. SHAP analysis and feature importance consistently identified urine output, age, LDL, BMI, and D-dimer as the top contributors. Compared with conventional ICU severity scores, the XGBoost model significantly outperformed APSIII (AUC = 0.661), LODs (AUC = 0.652), and OASIS (AUC = 0.647). The logistic regression-based nomogram achieved a C-index of 0.838, providing a user-friendly bedside tool for clinicians.

**Conclusion:**

The machine learning-based XGBoost model, incorporating ten readily available clinical variables, demonstrated excellent predictive performance for hypoalbuminemia in ICU patients with VTE and AKI. This model offers a practical and interpretable tool for early risk stratification and may guide proactive nutritional and therapeutic interventions in this vulnerable population.

## Introduction

Venous thromboembolism (VTE) and acute kidney injury (AKI) are two of the most frequent and serious complications encountered in the intensive care unit (ICU), with substantial morbidity and mortality burdens (1, 2). VTE, encompassing deep vein thrombosis and pulmonary embolism, affects approximately 10%–30% of critically ill patients and is associated with a threefold increase in mortality (3). AKI, occurring in up to 50% of ICU admissions, independently predicts poor outcomes, including progression to chronic kidney disease and increased healthcare costs (4). The coexistence of VTE and AKI creates a particularly vulnerable patient population characterized by hemodynamic instability, systemic inflammation, and metabolic derangements, predisposing them to a cascade of secondary complications.

Hypoalbuminemia, defined as serum albumin levels below 30–35 g/L, is a well-established marker of nutritional deficiency, systemic inflammation, and capillary leak syndrome in critically ill patients (5, 6). It serves as a powerful predictor of adverse outcomes, including prolonged mechanical ventilation, increased infection rates, longer ICU stays, and higher mortality (7, 8). In patients with VTE and AKI, hypoalbuminemia may exacerbate disease severity through multiple mechanisms: impaired drug binding (particularly anticoagulants and antibiotics), reduced oncotic pressure leading to tissue edema, compromised immune function, and exacerbation of prothrombotic states (9, 10). Despite its clinical significance, early identification of patients at risk of developing hypoalbuminemia remains challenging, as traditional clinical assessment often lacks predictive precision.

Conventional ICU severity scores, such as the Acute Physiology Score III (APSIII), Logistic Organ Dysfunction System (LODs), and Oxford Acute Severity of Illness Score (OASIS), provide valuable prognostic information for general ICU populations (11–13). However, these scores were primarily designed for mortality prediction and may not adequately capture the specific pathophysiological pathways leading to hypoalbuminemia in patients with VTE and AKI. Moreover, they often require extensive data collection and complex calculations, limiting their bedside utility for targeted nutritional risk assessment.

Machine learning (ML) has emerged as a powerful approach for developing clinical prediction models, offering the ability to identify complex, non-linear relationships among variables and improve predictive accuracy beyond traditional statistical methods (14, 15). Recent studies have successfully applied ML algorithms to predict various ICU outcomes, including AKI progression, sepsis mortality, and acute respiratory distress syndrome (16–18). However, to the best of our knowledge, no study has specifically developed an ML-based prediction model for hypoalbuminemia in ICU patients with coexisting VTE and AKI.

Therefore, the present study aimed to develop and validate a machine learning-based prediction model for hypoalbuminemia in this high-risk population. By leveraging LASSO regression for selection and comparing multiple ML algorithms, we sought to identify the optimal model and provide an interpretable, clinically applicable tool for early risk stratification.

## Materials and methods

### Study design and population

This retrospective study was conducted at the Affiliated Hospital of Jiangnan University (Wuxi, China). Consecutive ICU patients diagnosed with VTE and AKI between January 2021 and December 2025 were screened for inclusion. The study protocol was approved by the Institutional Ethics Committee, and the requirement for informed consent was waived due to the retrospective nature of the study.

Inclusion criteria were: (1) age >= 18 years; (2) admission to the ICU; (3) confirmed diagnosis of VTE (deep vein thrombosis or pulmonary embolism confirmed by imaging studies); (4) diagnosis of AKI according to the KDIGO criteria (increase in serum creatinine by >= 0.3 mg/dL within 48 h or >= 50% within 7 days, or urine output < 0.5 mL/kg/h for >= 6 h) (19); and (5) availability of complete clinical data.

Exclusion criteria were: (1) pregnancy or lactation; (2) ICU stay < 24 h; (3) pre-existing chronic hypoalbuminemia (albumin < 30 g/L at admission); (4) missing relevant variable data; (5) transfer from another ICU with prior VTE or AKI treatment.

### Data collection and outcome definition

A comprehensive set of clinical variables was collected within the first 24 h of ICU admission, including demographics [age, sex, height, weight, body mass index (BMI)], vital signs (heart rate, systolic blood pressure, diastolic blood pressure, mean arterial pressure, respiratory rate, temperature, SpO2), laboratory parameters (complete blood count, arterial blood gas, serum biochemistry, coagulation profiles, lipid profiles), comorbidities (Charlson comorbidity index and individual components), and therapeutic interventions [mechanical ventilation, continuous renal replacement therapy (CRRT), vasopressors, statins, diuretics, anticoagulants, ACE inhibitors, ARBs]. Urine output was recorded during the first 24 h.

The primary outcome was the occurrence of hypoalbuminemia during the ICU stay, defined as serum albumin < 30 g/L at any time after the first 24 h of admission. Patients who did not develop hypoalbuminemia during ICU stay were classified as the non-hypoalbuminemia group.

### Data preprocessing

Data preprocessing was performed using R (version 4.3.1). Categorical variables (e.g., sex, comorbidities, medication use) were encoded numerically. String-type variables (e.g., statin_drug, diuretic, anticoagulant, vasoactive_agents) were label-encoded using the LabelEncoder algorithm. All other continuous variables were retained in their original numerical form.

### Feature selection

To mitigate overfitting and enhance model interpretability, feature selection was performed using the least absolute shrinkage and selection operator (LASSO) logistic regression. Given the substantial heterogeneity in the scales of clinical variables (e.g., creatinine range: 60–1,851 vs. binary variables: 0–1), standardization was applied prior to LASSO regression using the StandardScaler method (mean = 0, standard deviation = 1). LASSO regression with 5-fold cross-validation was used to determine the optimal regularization parameter (lambda) that minimized the binomial deviance. Sort the absolute values of the standardized coefficients, and select the top 10 features with the strongest predictive signals for subsequent model development to balance model simplicity and reliability.

### Model development

Five machine learning algorithms were employed for model development: logistic regression (LR), random forest (RF), extreme gradient boosting (XGBoost), light gradient boosting machine (LightGBM), and support vector machine (SVM). All models were trained on the training cohort *n* = 409) and evaluated on the validation cohort *n* = 176).

Logistic Regression (LR): A classical linear model with L2 regularization and a maximum of 1,000 iterations. It served as the baseline interpretable model.
Random Forest (RF): An ensemble of 200 decision trees with a maximum depth of 8, using bootstrap sampling and random feature selection to reduce overfitting.
XGBoost: A gradient boosting framework with 200 estimators, a maximum depth of 4, a learning rate of 0.05, and the logloss evaluation metric. It was configured to handle class imbalance via scale_pos_weight adjustment.
LightGBM: A histogram-based gradient boosting algorithm with 200 estimators, a maximum depth of 4, and a learning rate of 0.05. It was optimized for large-scale data with leaf-wise tree growth.
Support Vector Machine (SVM): A radial basis function (RBF) kernel SVM with probability estimation enabled. It was trained on the selected features to assess the impact of feature scaling on kernel-based methods.

Hyperparameters were fixed across all models to ensure reproducibility and comparability.

### Model evaluation

Model performance was assessed using the following metrics: area under the receiver operating characteristic curve (AUC), accuracy, precision, recall, and F1-score. Five-fold stratified cross-validation was performed on the training cohort to evaluate internal stability. The primary metric for model selection was the validation AUC. Bootstrap resampling (2,000 iterations) was performed on the validation cohort to estimate the 95% confidence interval (CI) of the AUC for the optimal model.

To assess clinical utility, decision curve analysis (DCA) was performed for the best-performing model by calculating net benefit across a range of threshold probabilities (0–1). The model was compared against the “treat all” and “treat none” strategies.

SHapley Additive exPlanations (SHAP) analysis was applied to the optimal model to interpret the contribution of individual features to model predictions. Additionally, feature importance scores were extracted to quantify variable contributions.

### Comparison with conventional ICU scores

The predictive performance of the optimal machine learning model was compared with three conventional ICU severity scores: APSIII, LODs, and OASIS. These scores were calculated using standard definitions and evaluated as continuous predictors of hypoalbuminemia on the validation cohort.

### Statistical analysis

Continuous variables with normal distribution were expressed as mean ± standard deviation and compared between groups using the independent-samples t-test. Non-normally distributed continuous variables were reported as median with interquartile range [Q₁, Q₃] and analyzed using the Mann–Whitney U test. Categorical variables were summarized as frequencies and percentages (n, %) and compared using the chi-square test or Fisher's exact test, as appropriate. The variance inflation factor (VIF) was calculated to assess multicollinearity among the selected features, with VIF < 5 indicating no significant multicollinearity. A two-sided *P*-value < 0.05 was considered statistically significant. All analyses were performed using R (version 4.3.1).

## Results

### Baseline characteristics

A total of 585 ICU patients with VTE and AKI were included in the study, with 409 patients allocated to the training cohort and 176 to the validation cohort. The overall prevalence of hypoalbuminemia was 57.1% (334/585), with comparable rates in the training (56.5%) and validation (58.5%) cohorts. The baseline characteristics of patients with and without hypoalbuminemia are summarized in Table 1. There were statistically significant differences (*P* < 0.05) between the two groups in Apsiii, Lods, Oasis, Age, Height, Weight, BMI, Sbp, Dbp, MBP, Hemoglobin, Platelets, Hematocrit, Bicarbonate, Cr, ALP, Total Bilirubin, Ca, Inr, Pt, D D, LDL, Urineoutput, hypoalbuminemia, Tumor Cancer, Liver Disease, CRRT, anticoagulant drugs, vasoactive drugs, and Acei; whereas there were no statistically significant differences (*P* > 0.05) in Heart Rate, Gcs, Charlson Comorbidity Index, Dayicu, Resp Rate, Temperature, Spo2, Wbc, Basophil, Eosinophil, Lymphocyte, Monocyte, Neutrophil, Aniongap, Bun, ALT, AST, Na, K, Cl, Glucose, Ptt, TC, TG, HDL, Lactic Acid, Uric Acid, Sex, Statin Drug, Diuretic, Sirs, Diabetes, Hypertension, Myocardial Infarct, Congestive Heart Failure, Peripheral Vascular Disease, Cerebrovascular Disease, Chronic Pulmonary Disease, Obesity, Mechvent, Arb, Smoking, and Alcohol.

**Table 1:** The baseline characteristics of patients.

Variables	Total (*n* = 585)	Non-hypoproteinemia (*n* = 251)	Hypoproteinemia (*n* = 334)	P
Heart Rate, Mean ± SD	94.02 ± 17.65	93.07 ± 17.75	94.74 ± 17.56	0.257
Apsiii, M (Q₁, Q₃)	58.00 (47.00, 77.00)	53.00 (44.50, 66.00)	64.50 (48.25, 82.75)	<0.001
Lods, M (Q₁, Q₃)	7.00 (5.00, 10.00)	6.00 (5.00, 8.00)	7.00 (5.00, 10.00)	<.001
Oasis, M (Q₁, Q₃)	37.00 (30.00, 43.00)	34.00 (28.00, 40.00)	39.00 (32.00, 45.00)	<.001
Gcs, M (Q₁, Q₃)	15.00 (14.00, 15.00)	15.00 (14.00, 15.00)	15.00 (14.00, 15.00)	0.444
Charlson Comorbidity Index, M (Q₁, Q₃)	6.00 (3.00, 8.00)	5.00 (3.00, 8.00)	6.00 (3.25, 8.00)	0.250
Dayicu, M (Q₁, Q₃)	4.00 (2.00, 11.00)	4.00 (2.00, 10.50)	4.00 (2.00, 10.75)	0.865
Age, M (Q₁, Q₃)	69.00 (59.00, 79.00)	66.00 (52.50, 75.00)	72.00 (63.00, 82.00)	<.001
Height, M (Q₁, Q₃)	1.69 (1.63, 1.75)	1.70 (1.65, 1.76)	1.68 (1.63, 1.74)	0.039
Weight, M (Q₁, Q₃)	67.40 (59.60, 73.60)	68.60 (60.45, 74.65)	66.35 (59.12, 72.97)	0.042
BMI, M (Q₁, Q₃)	23.68 (21.50, 25.51)	23.04 (20.52, 24.73)	24.20 (21.93, 25.85)	<.001
Sbp, M (Q₁, Q₃)	110.00 (101.00,120.00)	112.00 (103.50,123.00)	109.00 (100.00,119.00)	0.007
Dbp, M (Q₁, Q₃)	63.00 (57.00, 69.00)	64.00 (57.00, 71.00)	61.50 (56.00, 67.00)	0.002
MBP, M (Q₁, Q₃)	76.00 (71.00, 83.00)	77.00 (72.00, 85.00)	75.00 (70.00, 81.00)	<.001
Resp Rate, M (Q₁, Q₃)	22.00 (19.00, 26.00)	22.00 (19.00, 25.00)	22.00 (19.00, 26.00)	0.076
Temperature, M (Q₁, Q₃)	36.80 (36.60, 37.20)	36.80 (36.60, 37.20)	36.80 (36.50, 37.10)	0.174
Spo2, M (Q₁, Q₃)	96.50 (94.90, 97.90)	96.20 (94.90, 97.80)	96.80 (94.90, 98.07)	0.209
Wbc, M (Q₁, Q₃)	13.60 (9.80, 18.90)	13.40 (10.00, 18.60)	14.05 (9.72, 19.00)	0.903
Hemoglobin, M (Q₁, Q₃)	102.50 (85.00, 119.00)	106.00 (88.25, 123.00)	99.00 (83.50, 116.75)	0.017
Platelets, M (Q₁, Q₃)	211.50 (175.50, 245.00)	218.00 (181.50, 251.25)	204.75 (172.00, 240.31)	0.010
Hematocrit, M (Q₁, Q₃)	32.10 (26.65, 37.15)	32.85 (27.23, 38.03)	31.08 (26.16, 36.54)	0.026
Basophil, M (Q₁, Q₃)	0.02 (0.00, 0.04)	0.02 (0.00, 0.04)	0.02 (0.00, 0.04)	0.365
Eosinophil, M (Q₁, Q₃)	0.01 (0.00, 0.09)	0.01 (0.00, 0.08)	0.01 (0.00, 0.09)	0.822
Lymphocyte, M (Q₁, Q₃)	1.00 (0.61, 1.55)	1.02 (0.61, 1.54)	1.00 (0.60, 1.55)	0.891
Monocyte, M (Q₁, Q₃)	0.67 (0.38, 1.10)	0.69 (0.41, 1.12)	0.65 (0.37, 1.09)	0.439
Neutrophil, M (Q₁, Q₃)	10.82 (6.81, 16.07)	10.71 (7.39, 15.77)	10.92 (6.54, 16.20)	0.806
Aniongap, M (Q₁, Q₃)	15.50 (13.00, 18.50)	15.50 (13.00, 18.00)	15.50 (13.00, 19.00)	0.863
Bicarbonate, M (Q₁, Q₃)	20.50 (17.50, 23.50)	21.00 (18.00, 24.00)	20.00 (16.50, 23.00)	0.001
Bun, M (Q₁, Q₃)	10.89 (7.68, 17.85)	10.53 (7.32, 16.42)	11.42 (8.07, 18.74)	0.061
Cr, M (Q₁, Q₃)	226.75 (166.28, 347.68)	196.51 (158.77, 275.90)	249.46 (189.00, 430.86)	<.001
ALT, M (Q₁, Q₃)	30.50 (17.00, 80.00)	28.50 (17.00, 71.75)	32.00 (17.00, 96.62)	0.357
AST, M (Q₁, Q₃)	49.00 (24.50, 129.00)	42.00 (25.00, 107.25)	54.00 (24.50, 150.88)	0.103
ALP, M (Q₁, Q₃)	93.00 (67.00, 135.00)	88.00 (62.00, 122.25)	98.75 (70.50, 146.88)	0.004
Total Bilirubin, M (Q₁, Q₃)	12.83 (6.84, 23.09)	11.97 (6.84, 19.10)	14.54 (7.70, 27.36)	0.024
Na, M (Q₁, Q₃)	138.00 (134.50, 141.50)	138.00 (134.50, 141.50)	137.75 (134.00, 141.50)	0.747
K, M (Q₁, Q₃)	4.40 (3.90, 4.90)	4.35 (3.90, 4.90)	4.43 (3.95, 4.90)	0.775
Ca, M (Q₁, Q₃)	8.15 (7.60, 8.65)	8.30 (7.70, 8.70)	8.07 (7.46, 8.60)	0.010
Cl, M (Q₁, Q₃)	103.00 (99.50, 106.50)	102.50 (99.25, 105.50)	103.50 (100.00, 107.00)	0.078
Glucose, M (Q₁, Q₃)	7.90 (6.28, 10.28)	8.07 (6.50, 10.44)	7.66 (6.19, 10.23)	0.279
Inr, M (Q₁, Q₃)	1.40 (1.25, 1.75)	1.35 (1.20, 1.58)	1.50 (1.30, 1.85)	<.001
Pt, M (Q₁, Q₃)	2.93 (2.70, 3.28)	2.88 (2.65, 3.17)	3.00 (2.73, 3.35)	0.004
Ptt, M (Q₁, Q₃)	37.90 (29.45, 80.45)	39.25 (28.95, 82.65)	37.85 (29.70, 75.84)	0.515
D D, M (Q₁, Q₃)	1.41 (0.58, 4.26)	1.01 (0.40, 2.59)	1.68 (0.76, 5.90)	<.001
TC, M (Q₁, Q₃)	4.21 (3.41, 5.15)	4.19 (3.40, 5.07)	4.23 (3.43, 5.17)	0.350
TG, M (Q₁, Q₃)	1.49 (1.03, 2.14)	1.50 (1.10, 2.09)	1.42 (1.00, 2.20)	0.903
HDL, M (Q₁, Q₃)	1.14 (0.93, 1.36)	1.13 (0.93, 1.33)	1.15 (0.94, 1.39)	0.181
LDL, M (Q₁, Q₃)	2.25 (1.65, 2.87)	2.04 (1.46, 2.61)	2.40 (1.78, 3.15)	<.001
Lactic Acid, M (Q₁, Q₃)	1.80 (1.30, 2.80)	1.80 (1.30, 2.60)	1.90 (1.30, 2.80)	0.153
Uric Acid, M (Q₁, Q₃)	321.30 (255.90, 416.50)	321.30 (261.80, 422.50)	321.30 (251.40, 412.92)	0.420
Urineoutput, M (Q₁, Q₃)	1,050.00 (485.00, 1,810.00)	1,400.00 (757.50, 2,282.50)	753.50 (337.50, 1,400.00)	<.001
Sex, *n*(%)				0.093
Male	324 (55.38)	149 (59.36)	175 (52.40)	
Female	261 (44.62)	102 (40.64)	159 (47.60)	
Statin Drug, *n*(%)				0.779
Atorvastatin	118 (20.17)	55 (21.91)	63 (18.86)	
None	390 (66.67)	164 (65.34)	226 (67.66)	
Pravastatin	26 (4.44)	9 (3.59)	17 (5.09)	
Rosuvastatin	17 (2.91)	7 (2.79)	10 (2.99)	
Simvastatin	34 (5.81)	16 (6.37)	18 (5.39)	
Diuretic, *n*(%)				0.610
Furosemide	275 (47.01)	124 (49.40)	151 (45.21)	
Spironolactone	73 (12.48)	29 (11.55)	44 (13.17)	
None	173 (29.57)	75 (29.88)	98 (29.34)	
Hydrochlorothiazide	32 (5.47)	13 (5.18)	19 (5.69)	
Acetazolamide	32 (5.47)	10 (3.98)	22 (6.59)	
Sirs, *n*(%)				0.273
0	4 (0.68)	1 (0.40)	3 (0.90)	
1	32 (5.47)	17 (6.77)	15 (4.49)	
2	103 (17.61)	49 (19.52)	54 (16.17)	
3	289 (49.40)	126 (50.20)	163 (48.80)	
4	157 (26.84)	58 (23.11)	99 (29.64)	
Diabetes, *n*(%)				0.845
No	415 (70.94)	177 (70.52)	238 (71.26)	
Yes	170 (29.06)	74 (29.48)	96 (28.74)	
Hypertension, *n*(%)				0.233
No	287 (49.06)	116 (46.22)	171 (51.20)	
Yes	298 (50.94)	135 (53.78)	163 (48.80)	
Myocardial Infarct, *n*(%)				0.419
No	476 (81.37)	208 (82.87)	268 (80.24)	
Yes	109 (18.63)	43 (17.13)	66 (19.76)	
Congestive Heart Failure, *n*(%)				0.158
No	396 (67.69)	162 (64.54)	234 (70.06)	
Yes	189 (32.31)	89 (35.46)	100 (29.94)	
Peripheral Vascular Disease, *n*(%)				0.331
No	533 (91.11)	232 (92.43)	301 (90.12)	
Yes	52 (8.89)	19 (7.57)	33 (9.88)	
Cerebrovascular Disease, *n*(%)				0.776
No	503 (85.98)	217 (86.45)	286 (85.63)	
Yes	82 (14.02)	34 (13.55)	48 (14.37)	
Chronic Pulmonary Disease, *n*(%)				0.082
No	436 (74.53)	178 (70.92)	258 (77.25)	
Yes	149 (25.47)	73 (29.08)	76 (22.75)	
Tumor Cancer, *n*(%)				<.001
No	411 (70.26)	201 (80.08)	210 (62.87)	
Yes	174 (29.74)	50 (19.92)	124 (37.13)	
Liver Disease, *n*(%)				0.027
No	472 (80.68)	213 (84.86)	259 (77.54)	
Yes	113 (19.32)	38 (15.14)	75 (22.46)	
Obesity, *n*(%)				0.493
No	460 (78.63)	194 (77.29)	266 (79.64)	
Yes	125 (21.37)	57 (22.71)	68 (20.36)	
CRRT, *n*(%)				0.015
No	490 (83.76)	221 (88.05)	269 (80.54)	
Yes	95 (16.24)	30 (11.95)	65 (19.46)	
Mechvent, *n*(%)				0.066
No	303 (51.79)	141 (56.18)	162 (48.50)	
Yes	282 (48.21)	110 (43.82)	172 (51.50)	
Anticoagulant, *n*(%)				<.001
No	95 (16.24)	12 (4.78)	83 (24.85)	
Yes	490 (83.76)	239 (95.22)	251 (75.15)	
Vasoactive drugs, *n*(%)				<.001
No	202 (34.53)	114 (45.42)	88 (26.35)	
Yes	383 (65.47)	137 (54.58)	246 (73.65)	
Acei, *n*(%)				0.011
No	492 (84.10)	200 (79.68)	292 (87.43)	
Yes	93 (15.90)	51 (20.32)	42 (12.57)	
Arb, *n*(%)				0.806
No	563 (96.24)	241 (96.02)	322 (96.41)	
Yes	22 (3.76)	10 (3.98)	12 (3.59)	
Smoking, n(%)				0.408
No	432 (73.85)	181 (72.11)	251 (75.15)	
Yes	153 (26.15)	70 (27.89)	83 (24.85)	
Alcohol, *n*(%)				0.611
No	359 (61.37)	157 (62.55)	202 (60.48)	
Yes	226 (38.63)	94 (37.45)	132 (39.52)	

### Feature selection

We randomly divided the dataset into a training set and a validation set at a ratio of 7:3, and the comparison between the training set and the validation set shown in Table 2. The results indicate that there are no significant differences between the training set and the validation set across all variables.

**Table 2:** Comparison between training set and validation set*.*

Variables	Total (*n* = 585)	Non-hypoproteinemia (*n* = 176)	Hypoproteinemia (*n* = 409)	*P*
Heart Rate, Mean ± SD	94.02 ± 17.65	93.52 ± 16.56	94.24 ± 18.11	0.654
Apsiii, M (Q₁, Q₃)	58.00 (47.00, 77.00)	57.00 (46.75, 73.00)	58.00 (47.00, 79.00)	0.271
Lods, M (Q₁, Q₃)	7.00 (5.00, 10.00)	7.00 (5.00, 9.00)	7.00 (5.00, 10.00)	0.117
Oasis, M (Q₁, Q₃)	37.00 (30.00, 43.00)	35.00 (29.75, 43.00)	37.00 (31.00, 44.00)	0.055
Gcs, M (Q₁, Q₃)	15.00 (14.00, 15.00)	15.00 (14.00, 15.00)	15.00 (14.00, 15.00)	0.983
Charlson Comorbidity Index, M (Q₁, Q₃)	6.00 (3.00, 8.00)	5.00 (3.00, 8.00)	6.00 (4.00, 8.00)	0.194
Dayicu, M (Q₁, Q₃)	4.00 (2.00, 11.00)	4.00 (2.00, 11.00)	4.00 (2.00, 10.00)	0.899
Age, M (Q₁, Q₃)	69.00 (59.00, 79.00)	71.00 (59.00, 78.00)	69.00 (60.00, 80.00)	0.917
Height, M (Q₁, Q₃)	1.69 (1.63, 1.75)	1.68 (1.62, 1.75)	1.70 (1.64, 1.75)	0.058
Weight, M (Q₁, Q₃)	67.40 (59.60, 73.60)	67.20 (58.85, 73.08)	67.40 (60.00, 74.00)	0.343
BMI, M (Q₁, Q₃)	23.68 (21.50, 25.51)	23.84 (21.53, 25.77)	23.53 (21.45, 25.39)	0.287
Sbp, M (Q₁, Q₃)	110.00 (101.00, 120.00)	110.00 (101.00, 120.00)	110.00 (102.00, 120.00)	0.727
Dbp, M (Q₁, Q₃)	63.00 (57.00, 69.00)	62.00 (56.00, 69.25)	63.00 (57.00, 69.00)	0.736
MBP, M (Q₁, Q₃)	76.00 (71.00, 83.00)	76.00 (71.00, 84.00)	76.00 (71.00, 83.00)	0.973
Resp Rate, M (Q₁, Q₃)	22.00 (19.00, 26.00)	22.00 (19.00, 26.00)	22.00 (19.00, 26.00)	0.427
Temperature, M (Q₁, Q₃)	36.80 (36.60, 37.20)	36.80 (36.60, 37.10)	36.80 (36.60, 37.20)	0.687
Spo2, M (Q₁, Q₃)	96.50 (94.90, 97.90)	96.55 (94.90, 97.80)	96.50 (94.90, 98.00)	0.929
Wbc, M (Q₁, Q₃)	13.60 (9.80, 18.90)	13.15 (9.90, 17.42)	14.30 (9.80, 19.50)	0.208
Hemoglobin, M (Q₁, Q₃)	102.50 (85.00, 119.00)	103.50 (85.88, 120.00)	101.50 (84.50, 119.00)	0.681
Platelets, M (Q₁, Q₃)	211.50 (175.50, 245.00)	214.12 (175.69, 242.88)	210.50 (175.50, 246.25)	0.964
Hematocrit, M (Q₁, Q₃)	32.10 (26.65, 37.15)	32.12 (26.69, 36.91)	32.00 (26.60, 37.40)	0.994
Basophil, M (Q₁, Q₃)	0.02 (0.00, 0.04)	0.02 (0.00, 0.04)	0.02 (0.00, 0.04)	0.840
Eosinophil, M (Q₁, Q₃)	0.01 (0.00, 0.09)	0.01 (0.00, 0.08)	0.01 (0.00, 0.09)	0.657
Lymphocyte, M (Q₁, Q₃)	1.00 (0.61, 1.55)	0.97 (0.56, 1.50)	1.03 (0.64, 1.55)	0.437
Monocyte, M (Q₁, Q₃)	0.67 (0.38, 1.10)	0.63 (0.40, 0.98)	0.68 (0.38, 1.16)	0.238
Neutrophil, M (Q₁, Q₃)	10.82 (6.81, 16.07)	10.54 (7.27, 14.50)	11.06 (6.75, 16.63)	0.693
Aniongap, M (Q₁, Q₃)	15.50 (13.00, 18.50)	15.00 (12.50, 18.12)	15.50 (13.00, 18.50)	0.147
Bicarbonate, M (Q₁, Q₃)	20.50 (17.50, 23.50)	20.50 (17.50, 23.50)	20.50 (17.50, 23.50)	0.827
Bun, M (Q₁, Q₃)	10.89 (7.68, 17.85)	11.96 (8.39, 17.72)	10.53 (7.50, 17.85)	0.362
Cr, M (Q₁, Q₃)	226.75 (166.28, 347.68)	222.99 (173.88, 366.57)	226.75 (166.28, 332.56)	0.632
ALT, M (Q₁, Q₃)	30.50 (17.00, 80.00)	28.50 (15.38, 78.88)	31.00 (17.50, 80.00)	0.289
AST, M (Q₁, Q₃)	49.00 (24.50, 129.00)	44.00 (23.88, 123.00)	52.00 (25.00, 132.00)	0.365
ALP, M (Q₁, Q₃)	93.00 (67.00, 135.00)	88.50 (65.38, 131.62)	94.00 (67.50, 137.00)	0.388
Total Bilirubin, M (Q₁, Q₃)	12.83 (6.84, 23.09)	11.97 (6.84, 23.09)	13.68 (7.70, 23.09)	0.591
Na, M (Q₁, Q₃)	138.00 (134.50, 141.50)	137.00 (133.88, 140.50)	138.00 (134.50, 142.00)	0.087
K, M (Q₁, Q₃)	4.40 (3.90, 4.90)	4.40 (3.95, 4.90)	4.40 (3.90, 4.90)	0.929
Ca, M (Q₁, Q₃)	8.15 (7.60, 8.65)	8.10 (7.60, 8.60)	8.20 (7.60, 8.70)	0.204
Cl, M (Q₁, Q₃)	103.00 (99.50, 106.50)	102.50 (100.00, 105.62)	103.50 (99.50, 106.50)	0.880
Glucose, M (Q₁, Q₃)	7.90 (6.28, 10.28)	8.18 (6.50, 10.13)	7.75 (6.22, 10.44)	0.424
Inr, M (Q₁, Q₃)	1.40 (1.25, 1.75)	1.40 (1.25, 1.75)	1.45 (1.25, 1.80)	0.581
Pt, M (Q₁, Q₃)	2.93 (2.70, 3.28)	2.93 (2.68, 3.24)	2.93 (2.70, 3.28)	0.619
Ptt, M (Q₁, Q₃)	37.90 (29.45, 80.45)	37.10 (28.89, 76.62)	38.30 (29.45, 81.50)	0.674
D D, M (Q₁, Q₃)	1.41 (0.58, 4.26)	1.46 (0.67, 4.48)	1.41 (0.55, 4.24)	0.424
TC, M (Q₁, Q₃)	4.21 (3.41, 5.15)	4.37 (3.40, 5.07)	4.14 (3.43, 5.15)	0.603
TG, M (Q₁, Q₃)	1.49 (1.03, 2.14)	1.57 (1.11, 2.12)	1.45 (1.00, 2.15)	0.440
HDL, M (Q₁, Q₃)	1.14 (0.93, 1.36)	1.12 (0.92, 1.34)	1.14 (0.96, 1.37)	0.384
LDL, M (Q₁, Q₃)	2.25 (1.65, 2.87)	2.30 (1.71, 2.92)	2.22 (1.65, 2.84)	0.280
Lactic Acid, M (Q₁, Q₃)	1.80 (1.30, 2.80)	1.80 (1.30, 2.60)	1.80 (1.30, 2.80)	0.262
Uric Acid, M (Q₁, Q₃)	321.30 (255.90, 416.50)	330.25 (261.80, 411.38)	321.30 (255.90, 416.50)	0.655
Urineoutput, M (Q₁, Q₃)	1,050.00 (485.00, 1,810.00)	1,174.50 (508.75, 1,876.25)	1,007.00 (470.00, 1,777.00)	0.243
Sex, *n*(%)				0.417
Male	324 (55.38)	93 (52.84)	231 (56.48)	
Female	261 (44.62)	83 (47.16)	178 (43.52)	
Statin Drug, *n*(%)				0.933
Atorvastatin	118 (20.17)	36 (20.45)	82 (20.05)	
None	390 (66.67)	114 (64.77)	276 (67.48)	
Pravastatin	26 (4.44)	9 (5.11)	17 (4.16)	
Rosuvastatin	17 (2.91)	5 (2.84)	12 (2.93)	
Simvastatin	34 (5.81)	12 (6.82)	22 (5.38)	
Diuretic, *n*(%)				0.283
Furosemide	275 (47.01)	90 (51.14)	185 (45.23)	
Spironolactone	73 (12.48)	16 (9.09)	57 (13.94)	
None	173 (29.57)	47 (26.70)	126 (30.81)	
Hydrochlorothiazide	32 (5.47)	12 (6.82)	20 (4.89)	
Acetazolamide	32 (5.47)	11 (6.25)	21 (5.13)	
Hypoproteinemia, *n*(%)				0.647
No	251 (42.91)	73 (41.48)	178 (43.52)	
Yes	334 (57.09)	103 (58.52)	231 (56.48)	
Sirs, *n*(%)				0.714
0	4 (0.68)	2 (1.14)	2 (0.49)	
1	32 (5.47)	7 (3.98)	25 (6.11)	
2	103 (17.61)	32 (18.18)	71 (17.36)	
3	289 (49.40)	85 (48.30)	204 (49.88)	
4	157 (26.84)	50 (28.41)	107 (26.16)	
Diabetes, *n*(%)				0.307
No	415 (70.94)	130 (73.86)	285 (69.68)	
Yes	170 (29.06)	46 (26.14)	124 (30.32)	
Hypertension, *n*(%)				0.335
No	287 (49.06)	81 (46.02)	206 (50.37)	
Yes	298 (50.94)	95 (53.98)	203 (49.63)	
Myocardial Infarct, *n*(%)				0.330
No	476 (81.37)	139 (78.98)	337 (82.40)	
Yes	109 (18.63)	37 (21.02)	72 (17.60)	
Congestive Heart Failure, *n*(%)				0.117
No	396 (67.69)	111 (63.07)	285 (69.68)	
Yes	189 (32.31)	65 (36.93)	124 (30.32)	
Peripheral Vascular Disease, *n*(%)				0.910
No	533 (91.11)	160 (90.91)	373 (91.20)	
Yes	52 (8.89)	16 (9.09)	36 (8.80)	
Cerebrovascular Disease, *n*(%)				0.141
No	503 (85.98)	157 (89.20)	346 (84.60)	
Yes	82 (14.02)	19 (10.80)	63 (15.40)	
Chronic Pulmonary Disease, *n*(%)				0.091
No	436 (74.53)	123 (69.89)	313 (76.53)	
Yes	149 (25.47)	53 (30.11)	96 (23.47)	
Tumor Cancer, *n*(%)				0.391
No	411 (70.26)	128 (72.73)	283 (69.19)	
Yes	174 (29.74)	48 (27.27)	126 (30.81)	
Liver Disease, *n*(%)				0.820
No	472 (80.68)	143 (81.25)	329 (80.44)	
Yes	113 (19.32)	33 (18.75)	80 (19.56)	
Obesity, *n*(%)				0.456
No	460 (78.63)	135 (76.70)	325 (79.46)	
Yes	125 (21.37)	41 (23.30)	84 (20.54)	
CRRT, *n*(%)				0.172
No	490 (83.76)	153 (86.93)	337 (82.40)	
Yes	95 (16.24)	23 (13.07)	72 (17.60)	
Mechvent, *n*(%)				0.217
No	303 (51.79)	98 (55.68)	205 (50.12)	
Yes	282 (48.21)	78 (44.32)	204 (49.88)	
Anticoagulant, *n*(%)				0.528
No	95 (16.24)	26 (14.77)	69 (16.87)	
Yes	490 (83.76)	150 (85.23)	340 (83.13)	
Vasoactive drugs, *n*(%)				0.541
No	202 (34.53)	64 (36.36)	138 (33.74)	
Yes	383 (65.47)	112 (63.64)	271 (66.26)	
Acei*, n*(%)				0.809
No	492 (84.10)	149 (84.66)	343 (83.86)	
Yes	93 (15.90)	27 (15.34)	66 (16.14)	
Arb, *n*(%)				0.443
No	563 (96.24)	171 (97.16)	392 (95.84)	
Yes	22 (3.76)	5 (2.84)	17 (4.16)	
Smoking, *n*(%)				0.842
No	432 (73.85)	129 (73.30)	303 (74.08)	
Yes	153 (26.15)	47 (26.70)	106 (25.92)	
Alcohol, *n*(%)				0.460
No	359 (61.37)	112 (63.64)	247 (60.39)	
Yes	226 (38.63)	64 (36.36)	162 (39.61)	

Standardized LASSO regression with 5-fold cross-validation identified ten optimal features for model development. The standardized coefficients and their absolute values are presented in Table 3. The selected predictors included creatinine (cr; coefficient = 0.802), age (0.725), tumor/cancer (0.638), BMI (0.627), LDL (0.550), urine output (0.487), D-dimer (0.459), Charlson comorbidity index (0.406), anticoagulant use (0.307), and ALT (0.281). Notably, creatinine and age emerged as the strongest independent predictors in the linear model, while urine output and BMI demonstrated substantial contributions in the non-linear tree-based models.

**Table 3:** Standardized LASSO coefficients of the selected top 10 features.

Rank	Feature	Standardized Coefficient	Absolute Value
1	Creatinine (cr)	0.802	0.802
2	Age	0.725	0.725
3	Tumor/Cancer	0.638	0.638
4	BMI	0.627	0.627
5	LDL	0.550	0.550
6	Urine Output	0.487	0.487
7	D-dimer (D_D)	0.459	0.459
8	Charlson Comorbidity Index	0.406	0.406
9	Anticoagulant Use	0.307	0.307
10	ALT	0.281	0.281

### Multicollinearity assessment

The multicollinearity assessment revealed no significant collinearity among the selected features, with all VIF values below 1.6 (highest: Charlson comorbidity index, VIF = 1.53; Table 4).

**Table 4:** Variance inflation factor (VIF) of the selected top 10 features.

Feature	VIF
Charlson comorbidity index	1.53
Tumor/Cancer	1.43
Age	1.14
Urine output	1.12
Anticoagulant use	1.11
D-dimer	1.08
Creatinine	1.06
LDL	1.05
ALT	1.05
BMI	1.04

### Model performance and comparison

The performance comparison results of the five machine learning models on the validation cohort are shown in Table 5. The XGBoost model performed best, with a5-fold cross-validation AUC of 0.901 ± 0.042 and a validation set AUC of 0.882, while its Accuracy, Recall, and F1-Score were 0.784, 0.816, 0.816, and 0.816 respectively; Random Forest (RF) and LightGBM followed, with validation AUCs of 0.878 and 0.870, respectively, and similar across all metrics; Logistic Regression (LR) achieved a validation AUC of 0.838, showing acceptable performance; whereas Support Vector Machine (SVM), being sensitive to unstandardized feature scales due the RBF kernel, yielded a validation AUC of only 0.662, significantly inferior to the other models. Overall, except for SVM, the performance differences between crossvalidation and the validation set for all models were small, suggesting good generalization ability and no obvious overfitting.

**Table 5:** Performance of the five machine learning models on the validation cohort.

Model	CV AUC (mean ± SD)	Test AUC	Accuracy	Precision	Recall	F1-Score
LR	0.873 ± 0.044	0.838	0.761	0.785	0.816	0.800
RF	0.892 ± 0.045	0.878	0.767	0.804	0.796	0.800
XGBoost	0.901 ± 0.042	0.882	0.784	0.816	0.816	0.816
LightGBM	0.892 ± 0.044	0.870	0.784	0.816	0.816	0.816
SVM	0.720 ± 0.059	0.662	0.614	0.670	0.670	0.670

### XGBoost performance

XGBoost achieved the highest validation AUC (0.882), outperforming RF (0.878), LightGBM (0.870), LR (0.838), and SVM (0.662). The 5-fold cross-validation AUC for XGBoost was 0.901 ± 0.042, with a minimal difference between CV and test performance (−0.019), indicating excellent generalizability without significant overfitting. Bootstrap resampling (2,000 iterations) confirmed the stability of the XGBoost model, yielding a 95% CI of 0.832–0.924 for the validation AUC.

The receiver operating characteristic (ROC) curves for all five models are presented in Figure 1. XGBoost demonstrated the most favorable curve, with superior sensitivity and specificity across most threshold values. The SVM model showed markedly inferior performance, which was attributed to the sensitivity of the RBF kernel to unscaled feature magnitudes.

**Figure 1:**
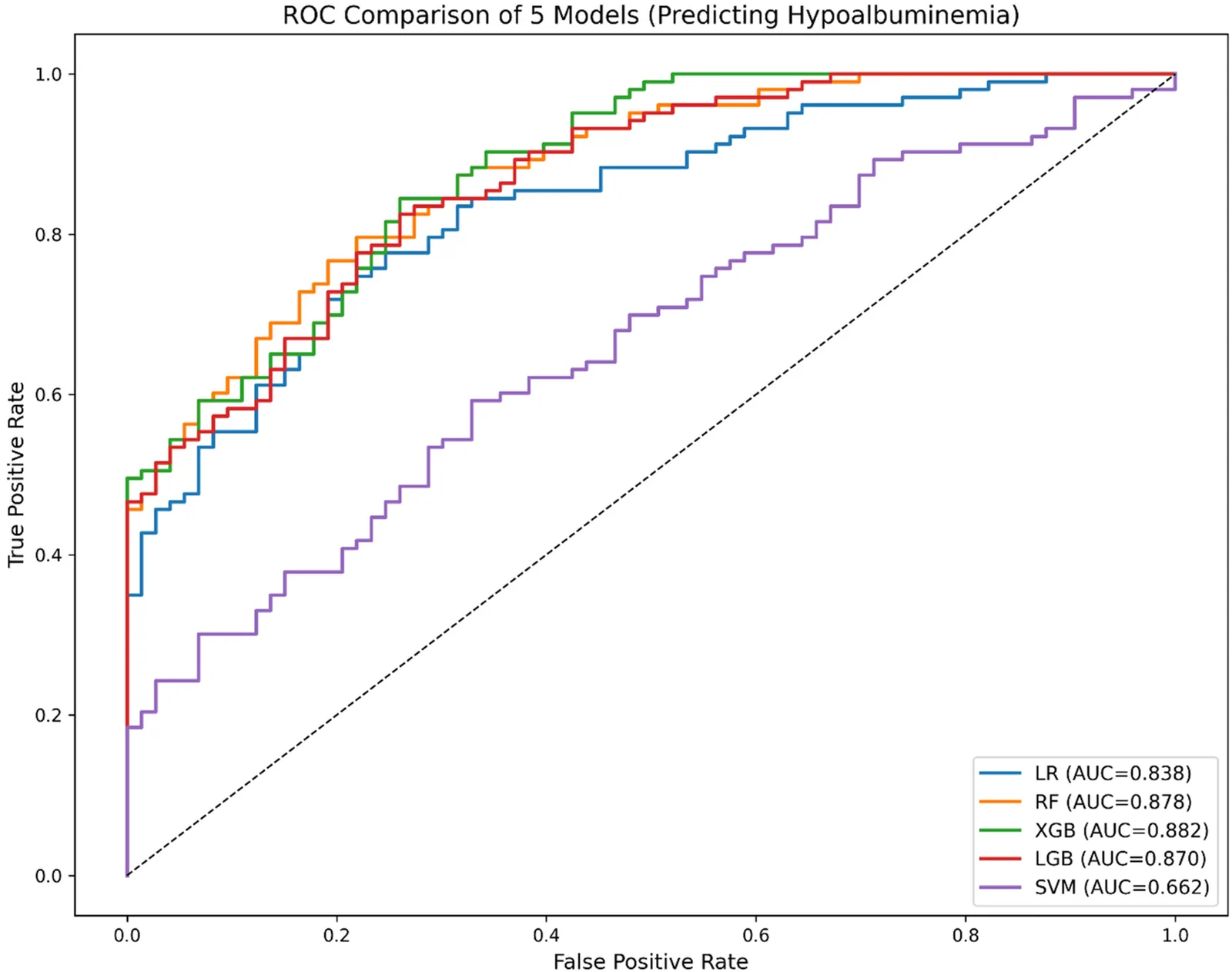
Compare the ROC curves of the five models.

### Decision curve analysis

Decision curve analysis of the XGBoost model demonstrated a positive net benefit across a broad range of threshold probabilities (0.1–0.9), compared to both the “treat all” and “treat none” strategies (Figure 2). The model offered the highest clinical utility at threshold probabilities between 0.2 and 0.6, which are clinically relevant for initiating nutritional interventions and albumin supplementation in ICU patients.

**Figure 2:**
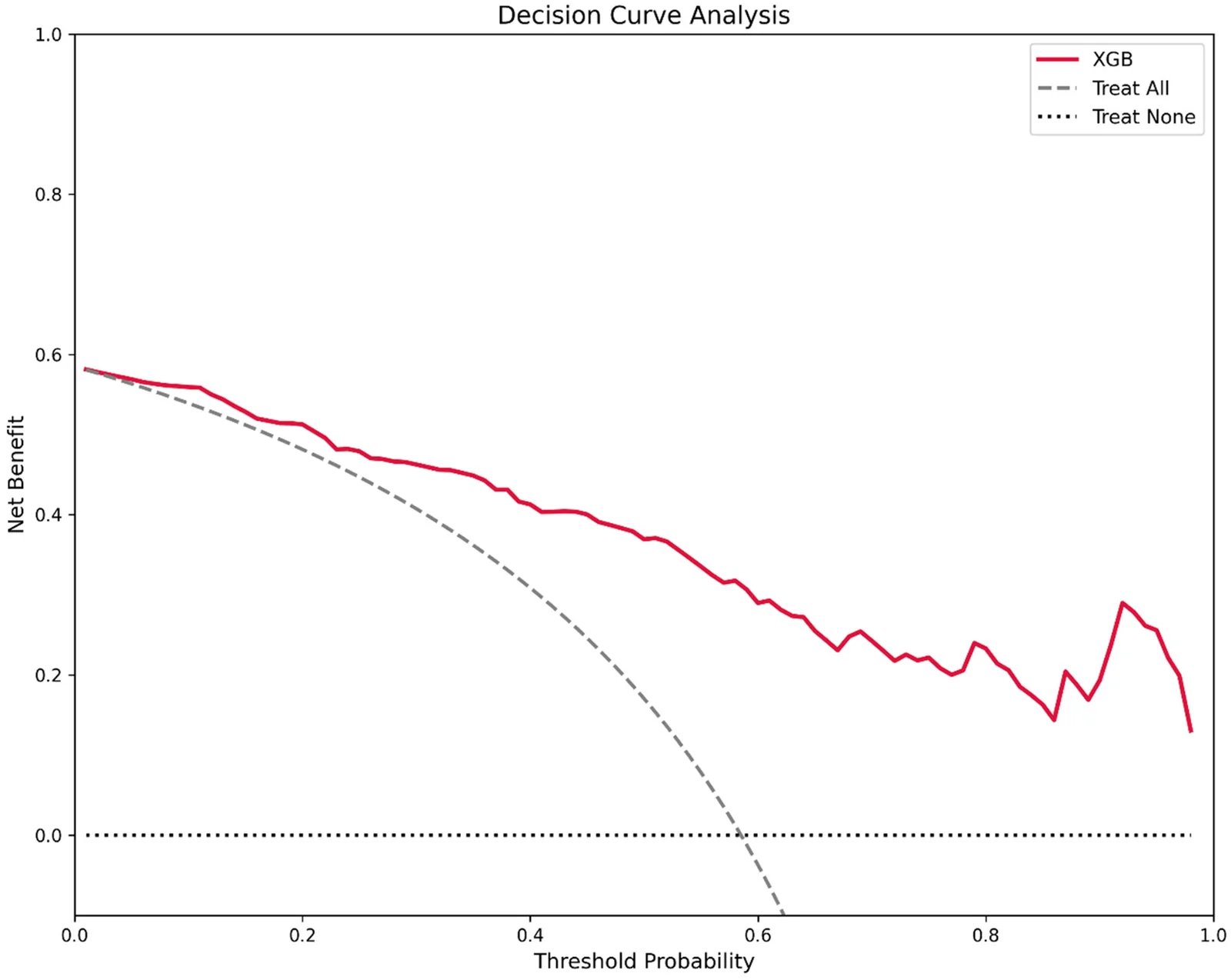
DCA curve.

### Feature importance and model interpretability

The feature importance scores from the XGBoost model are illustrated in Figure 3. The top five contributors were urine output (importance = 0.141), age (0.137), LDL (0.127), BMI (0.124), and D-dimer (0.116). Creatinine, which ranked first in the LASSO linear model, was ranked seventh in XGBoost (0.093), suggesting that its predictive effect may be partially mediated through interactions with other variables (e.g., urine output and BMI) in the non-linear model.

**Figure 3:**
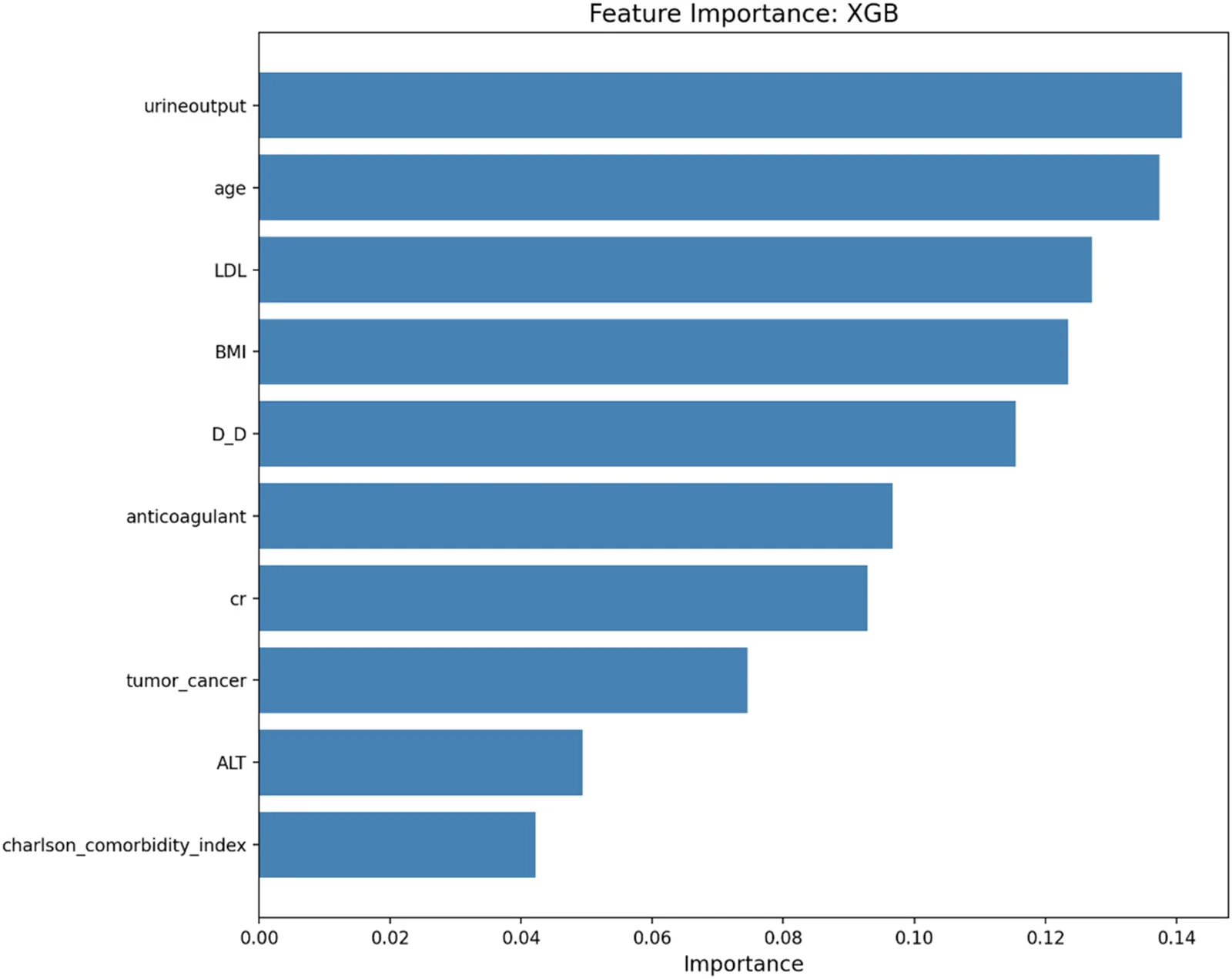
The feature importance scores from the XGBoost model.

SHAP summary analysis (Figure 4) further elucidated the directional effects of individual features. Higher urine output and higher BMI were associated with increased SHAP values (positive contribution to hypoalbuminemia risk), consistent with the pathophysiology of fluid overload and metabolic dysfunction in VTE-AKI patients. Advanced age, elevated D-dimer, and tumor presence also consistently increased the predicted risk, while anticoagulant use showed a more nuanced pattern depending on the clinical context.

**Figure 4:**
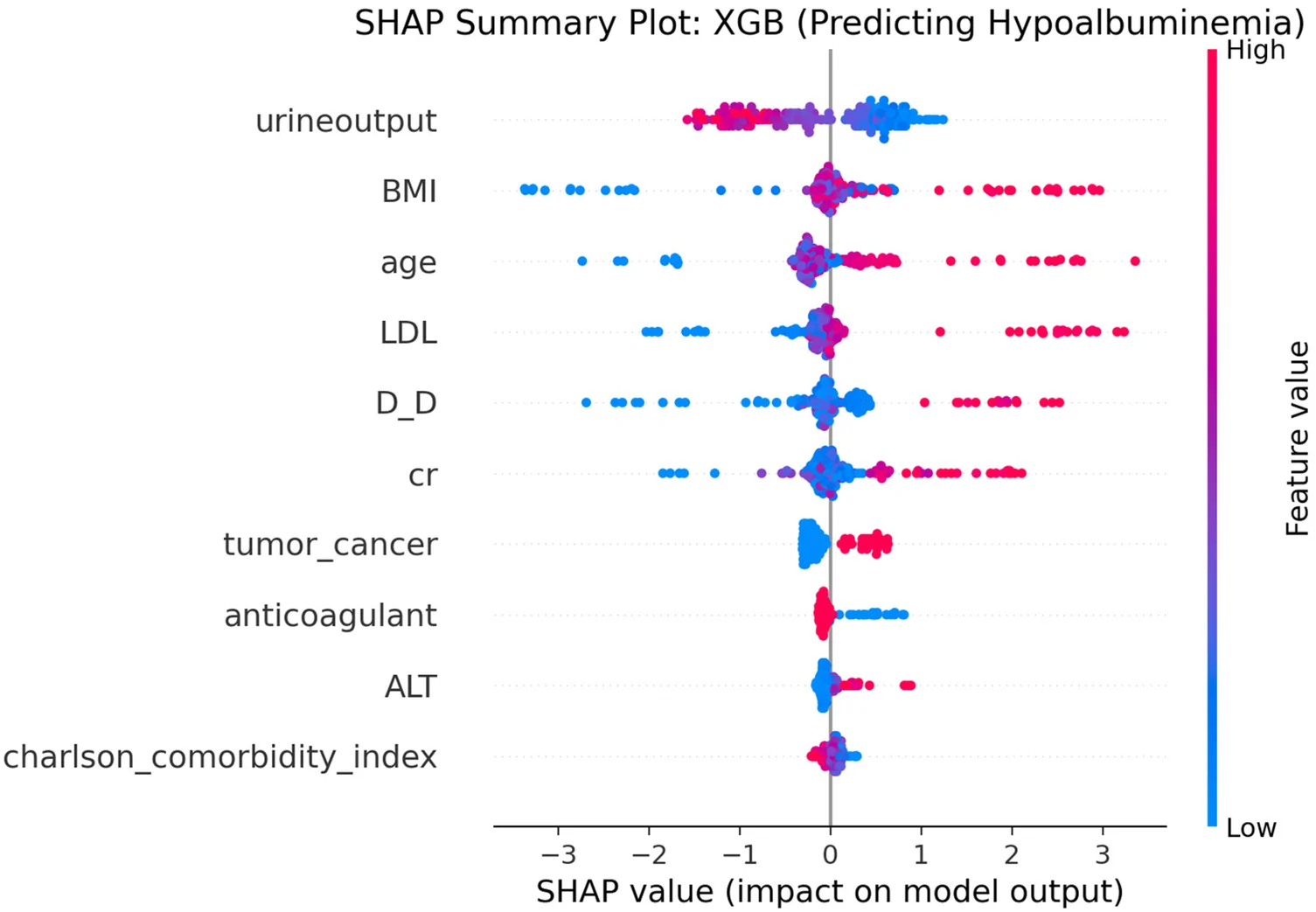
SHAP summary analysis.

### Comparison with conventional ICU scores

The predictive performance of the XGBoost model was benchmarked against three established ICU severity scores (Figure 5). The XGBoost model (AUC = 0.882) significantly outperformed APSIII (AUC = 0.661), LODs (AUC = 0.652), and OASIS (AUC = 0.647). This finding underscores the limitation of generic severity scores for specific complication prediction and highlights the incremental value of the machine learning approach tailored for hypoalbuminemia risk in this population.

**Figure 5:**
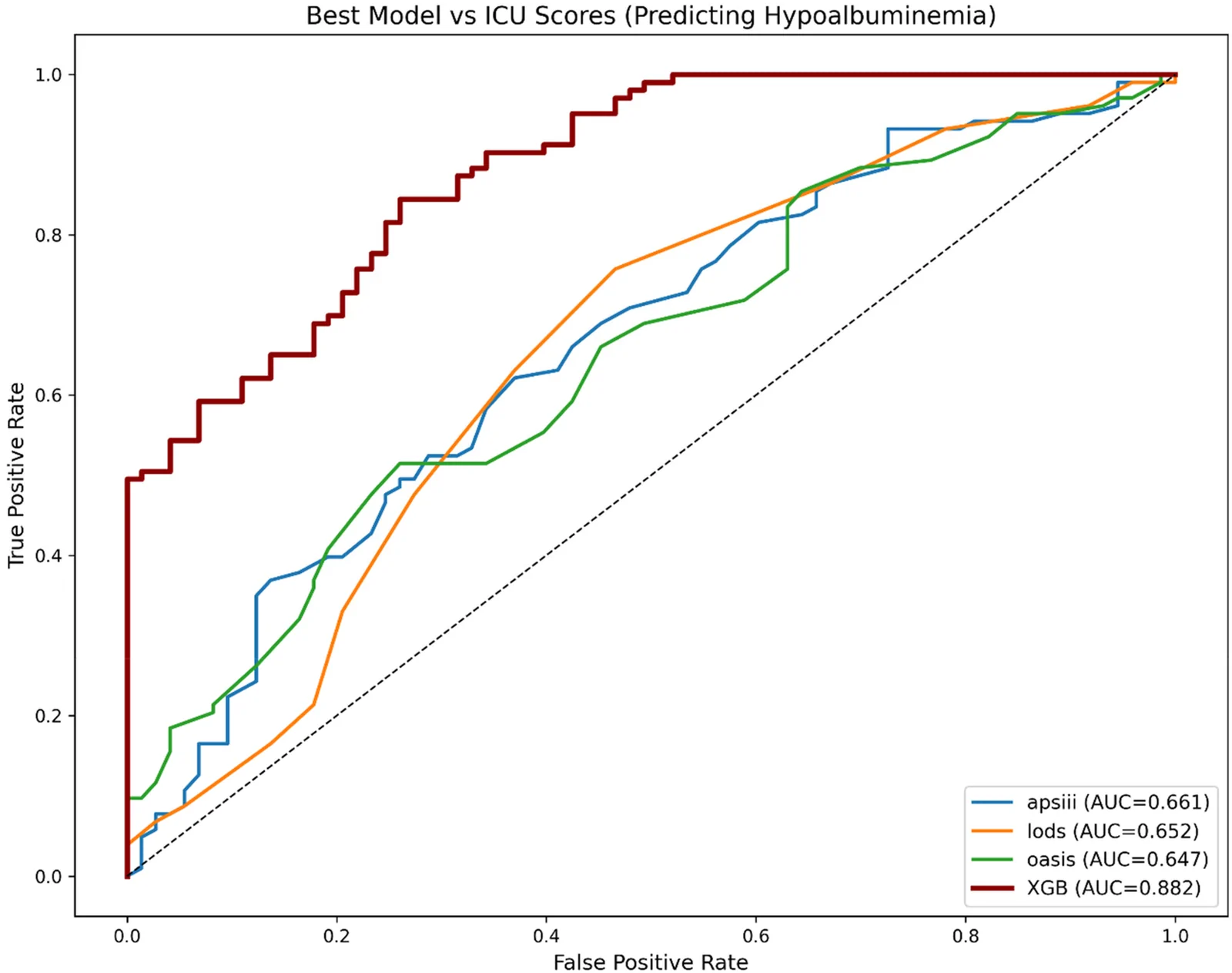
XGBoost compared with conventional ICU scores*.*

## Discussion

In this retrospective study of 585 ICU patients with coexisting VTE and AKI, we developed and validated a machine learning-based prediction model for hypoalbuminemia. The XGBoost algorithm, incorporating ten readily available clinical variables, achieved the highest predictive accuracy (AUC = 0.882, 95% CI: 0.832–0.924) on an independent validation cohort. The model demonstrated favorable clinical utility on decision curve analysis and significantly outperformed conventional ICU severity scores (APSIII, LODs, and OASIS). To our knowledge, this is the first study to specifically develop an ML-based prediction model for hypoalbuminemia in this high-risk population.

### Key predictors and pathophysiological insights

The selected top 10 predictors reflect the multifactorial etiology of hypoalbuminemia in patients with VTE and AKI. Creatinine and urine output emerged as critical indicators, reflecting the severity of renal dysfunction and fluid balance. In AKI, reduced glomerular filtration leads to impaired clearance of inflammatory mediators and uremic toxins, which can suppress hepatic albumin synthesis and increase vascular permeability (20, 21). Concurrently, fluid overload-often manifesting as increased urine output in the diuretic phase or oliguria in the oliguric phase-alters albumin distribution and dilution, complicating the clinical picture.

Age was consistently identified as a strong predictor across all models. Elderly patients exhibit reduced hepatic synthetic reserve, decreased nutritional intake, and higher baseline inflammation, all of which predispose to albumin depletion (22). BMI and LDL provided metabolic insights: obesity is associated with chronic low-grade inflammation and altered protein metabolism, while dyslipidemia (reflected by LDL) may indicate underlying metabolic syndrome and endothelial dysfunction, both of which are linked to hypoalbuminemia (23, 24).

The inclusion of tumor/cancer and Charlson comorbidity index underscores the role of chronic disease burden. Malignancy directly increases catabolic demand and reduces albumin synthesis via pro-inflammatory cytokines (IL-6, TNF-alpha) (25). D-dimer, a marker of fibrinolysis and thrombotic burden, was also predictive, likely reflecting the hypercoagulable and inflammatory milieu of VTE, which synergizes with AKI-induced systemic inflammation to exacerbate albumin loss (26).

ALT served as a proxy for hepatic function, as impaired hepatocellular integrity directly reduces albumin production. The inclusion of anticoagulant use was clinically relevant, as anticoagulation therapy may be associated with bleeding events or heparin-induced thrombocytopenia, both of which can complicate nutritional status and inflammatory responses in ICU patients (27).

### Model selection and performance

The superior performance of XGBoost (AUC = 0.882) over LR (0.838) and SVM (0.662) can be attributed to its ability to capture non-linear interactions and handle heterogeneous feature scales. XGBoost's gradient boosting framework sequentially corrects errors through shallow trees, effectively modeling complex relationships (e.g., the interaction between creatinine and urine output) without explicit feature engineering (28). RF performed comparably (AUC = 0.878), consistent with its ensemble nature and robustness to outliers. LightGBM, despite its computational efficiency, showed slightly lower validation performance (AUC = 0.870), possibly due to its leaf-wise growth strategy being more prone to overfitting on smaller datasets (29).

The SVM's poor performance (AUC = 0.662) was expected, as the RBF kernel is highly sensitive to feature scaling. With creatinine and urine output spanning three orders of magnitude compared to binary variables, the kernel distance metric was dominated by large-scale features, obscuring the contributions of categorical predictors. While standardizing the top 10 features could improve SVM performance, this limitation highlights an important practical consideration: tree-based models are inherently more robust to heterogeneous clinical data scales and require less preprocessing, making them preferable for ICU prediction tasks (30).

### Clinical implications

The present model offers several clinical advantages. First, all ten predictors are routinely available within the first 24 h of ICU admission, enabling early risk stratification without additional costs or specialized testing. Second, the model significantly outperformed generic ICU severity scores (APSIII, LODs, OASIS), suggesting that disease-specific prediction is more informative than general severity assessment for targeted nutritional interventions.

Early identification of high-risk patients is particularly important in VTE-AKI populations. Hypoalbuminemia exacerbates anticoagulant pharmacokinetics (reduced protein binding of warfarin and heparin), increases bleeding risk, impairs wound healing, and prolongs ICU stay (31). Proactive intervention guided by this model may improve patient outcomes, reduce complications, and optimize resource allocation.

### Limitations

Several limitations should be acknowledged. First, this was a single-center, retrospective study, which may limit generalizability to other institutions or populations. External validation using multi-center data is warranted before widespread clinical adoption. Second, the dataset lacked explicit temporal information (e.g., exact time of hypoalbuminemia onset), precluding time-to-event analysis and dynamic risk assessment. Future studies should incorporate time-varying covariates and recurrent event models. Third, albumin levels were not directly available in the dataset; the hypoalbuminemia status was provided as a binary outcome. While this simplifies the prediction task, it precludes analysis of albumin concentration trends. Fourth, the sample size (*n* = 585), while adequate for initial model development, may be insufficient for deep learning or very high-dimensional feature spaces. Fifth, the SVM model was trained on unscaled features, which may have underestimated its true performance. Finally, the model does not establish causality; it identifies associations that require prospective interventional studies to confirm clinical utility.

### Future directions

Future research should focus on: (1) external validation in independent, multi-center cohorts to confirm generalizability; (2) prospective clinical trials evaluating whether risk-stratified nutritional interventions based on the model improve outcomes; (3) integration of time-series data (e.g., repeated laboratory measurements) to develop dynamic prediction models; (4) comparison with deep learning architectures (e.g., recurrent neural networks, transformers) on larger datasets; and (5) implementation studies assessing the real-world impact of the nomogram on clinical decision-making and patient outcomes.

## Conclusion

We developed and validated a machine learning-based prediction model for hypoalbuminemia in ICU patients with VTE and AKI. The XGBoost model, incorporating ten routinely available clinical variables, demonstrated excellent predictive performance (AUC = 0.882) and favorable clinical utility. The model significantly outperformed conventional ICU severity scores and was translated into a user-friendly nomogram (C-index = 0.838). This tool may facilitate early risk stratification and guide proactive nutritional and therapeutic interventions in this vulnerable population. External validation and prospective clinical studies are warranted to confirm its clinical utility.

## Data Availability

The raw data supporting the conclusions of this article will be made available by the authors, without undue reservation.
